# Cone ERG Changes During Light Adaptation in Two All-Cone Mutant Mice: Implications for Rod-Cone Pathway Interactions

**DOI:** 10.1167/iovs.19-27242

**Published:** 2019-08

**Authors:** Ronald A. Bush, Atsuhiro Tanikawa, Yong Zeng, Paul A. Sieving

**Affiliations:** 1 National Institute on Deafness and Other Communication Disorders, National Institutes of Health, Bethesda, Maryland, United States; 2 Department of Ophthalmology, Fujita Health University School of Medicine, Toyoake, Aichi, Japan; 3 National Eye Institute, National Institutes of Health, Bethesda, Maryland, United States

**Keywords:** electroretinogram, rod-cone interaction, light adaptation, cones, oscillatory potentials

## Abstract

**Purpose:**

The b-wave of the cone ERG increases in amplitude and speed during the first few minutes of adaptation to a rod-suppressing background light. Earlier studies implicate rod pathway input to the cone pathway in these changes.

**Methods:**

The timing and amplitude of the cone b-wave and isolated oscillatory potentials (OP) during the first 10 minutes of light adaptation in wild-type (WT) mice and two mutant lines without functional rods was examined: rhodopsin knockout (*Rho^−/−^*), lacking rod outer segments, and NRL knockout (*Nrl^−/−^*), in which rods are replaced by S-cones. Expression of the immediate-early gene *c-fos*, which is increased in the inner retina by light-induced activity, was evaluated by immunohistochemistry in dark- and light-adapted retinas.

**Results:**

WT b-wave and OP amplitudes increased, and implicit times decreased during light adaptation. Subtracting OP did not alter b-wave changes. *Rho^−/−^* b-wave and OP amplitudes did not increase during adaptation. B-wave timing and amplitude and the timing of the major OP at 1 minute of adaptation were equivalent to WT at 10 minutes. The light-adapted ERG b-wave in *Nrl^−/−^* mice, which originates in both the rod and cone pathways, changed in absolute amplitude and timing similar to WT. *C-fos* expression was present in the inner retinas of dark-adapted *Rho^−/−^* but not WT or *Nrl^−/−^* mice.

**Conclusions:**

Activity in the distal rod pathway produces changes in the cone ERG during light adaptation. Rods in *Rho^−/−^* mice constitutively activate this rod-cone pathway interaction. The rod pathway S-cones in *Nrl^−/−^* mice may maintain the WT interaction.

Visual signals in the retina traverse two distinct pathways: the rod pathway with photoreceptors and downstream signaling specialized for dim light and high sensitivity vision, and the cone pathway, which is specialized for brighter light and color vision. Many studies have shown that there is crosstalk between the two pathways, particularly from rods to cones, at the level of photoreceptor and retinal interneurons. It is difficult to detect effects of this interaction on visual function under normal conditions. Strategies that have been used include comparing the responses of cones at different degrees of rod activation or using drugs to block signaling between^1^ or within the pathways.^2^

Imposing a weak, steady background-adapting light can saturate the rods and suppress their response to incremental stimulus changes, which allows cone pathway responses to be analyzed in relative isolation. During the first few minutes of adaptation to a rod-saturating background light, the cone ERG b-wave amplitude increases, and the time to peak, or implicit time, decreases in animals^3^,^4^ and in humans.^5^–^10^ Some animal studies suggested that rod signaling mediates these changes in the cone ERG (Schiavone MT, Peachey NS. *IOVS* 2002;43:ARVO E-Abstract 1840).^1^,^3^ Support for a role of the rod pathway in modulating human cone pathway responses comes from experiments showing that cone threshold sensitivity decreases as rod sensitivity increases during dark adaptation,^11^ and from multifocal ERG changes during light adaptation, which are greater outside the central retina where rod density is higher.^12^ However, other evidence from humans suggests that cone ERG adaptation is mediated directly by the cones themselves or by the cone retinal circuitry.^9^,^10^,^13^

The development of gene knockout mice without functioning rods allows study of the cone pathway response to background light in the absence of the rod response. Previous studies revealed that some cone ERG changes in early light adapation are absent in transducin knockout mice^14^ (Schiavone MT, Peachey NS. *IOVS* 2002;43:ARVO E-Abstract 1840), and ex vivo experiments in wild-type (WT) mouse retina support a role for the rod pathway in producing these changes.^1^ Rhodopsin knockout (*Rho^−/−^*)^15^ and neural retina leucine zipper knockout (*Nrl^−/−^*)^16^ mice both lack functional rods but have normal cone and cone pathway function.^17^,^18^ These two models are very different, however, in their photoreceptor content and coupling to the inner retina. The *Rho^−/−^* mouse has a full complement of rods and cones at 3 to 4 weeks of age^15^ (Bush et al., unpublished observations, 1999) the time of retinal maturity, but rods lack outer segments that contain visual pigment. Rods gradually decline in number to approximately 15% by 75 days, but cone cell numbers in *Rho*^−/−^ remain relatively stable up to this age (Bush et al., unpublished observations, 1999). Cone ERG responses steadily decline to unrecordable levels between 45 and 80 days.^17^ Rod pathway morphology and rod synaptic contacts remain normal up to 7 weeks.^19^ In *Nrl^−/−^* retina, rods are replaced by short-wavelength cones (S-cones) that make synaptic contacts with the rod pathway that otherwise appears anatomically and immunohistochemically normal.^20^ Thus, the rod pathway in both models is intact but has no rod activity elicited in response to light. If rod-mediated responses to saturating background light are important for the amplitude and timing changes in cone pathway responses during light adaptation, then the ERG of these cone-only retinas should not exhibit these characteristic changes.

## Materials and Methods

### Animals

*Nrl*^−/−16^ and *Rho*^−/−15^ mice were propagated from the original lines on which we previously reported. ERGs were recorded from both eyes of 6-month-old male WT (*Nrl^+/+^*), 6-month-old male *Nrl*^−/−^, and 4-week-old male *Rho*^−/−^ mice. Experiments were conducted in accord with the ARVO Statement for the Use of Animals in Ophthalmic and Vision Research, and the protocols were approved by the Animal Care and Use Committee of the National Eye Institute, National Institutes of Health.

### ERG Recordings

Animals were dark adapted overnight and then prepared under dim red light. They were anesthetized with ketamine (80 mg/kg) and xylazine (4 mg/kg) intraperitoneally, and pupils were dilated with 0.1% atropine and 0.1% phenylephrine HCl. Body temperature was maintained near 38°C with a heating pad. The ERG was recorded with gold-wire loop active electrode placed on the center of the cornea, and gold-wire reference electrodes touching the sclera near the limbus. Tetracaine 1% was used as a topical anesthesia, and 2% methylcellulose was used as a corneal lubricant and to ensure good electrode contact. The electrodes were made in our laboratory from 0.1-mm-diameter gold wire with a 1.0-mm loop at one end as in previous studies.^21^ A reference ground wire was attached to the ear. Signals were amplified 5000 times and frequency limited to 0.1 to 1000 Hz using a DAM50 amplifier (World Precision Instruments, Sarasota, FL, USA) and 60-Hz analog notch filter and then were digitized at 5-kHz rate with 12-bit resolution.

Full-field 30-μs flashes from a xenon photostrobe lamp (PS33 Photic Stimulator; Grass Instrument Co., West Warwick, RI, USA) were presented in a 30-cm sphere to dark-adapted and light-adapted mice at luminances from threshold up to a maximum of 0.6 log cd·s/m^2^. A 1.6-log cd/m^2^ white background supplied by a tungsten lamp was used to record the cone ERG by fully suppressing the rod contribution even at maximum stimulus luminance. We considered using a weaker background that might suppress only the rods without affecting cones. However, we found that even the dimmest background that suppressed the WT rod contribution slightly elevated the cone b-wave threshold in *Rho^−/−^* mice,^17^ indicating it may not be possible to completely isolate rod and cone effects by this method. In the dark-adapted state, up to 15 responses were averaged with an interstimulus interval (ISI) of 3 to 30 seconds depending on stimulus luminance. In the light-adapted state, 20 responses with an ISI of 1 second were averaged. To investigate changes during light adaptation following overnight dark adaptation, the 0.6 log cd·s/m^2^ stimulus flashes were presented beginning 10 seconds after the background light was turned on (time = 0 minutes) for the first recording and then again at 1-minute intervals for the subsequent 10 minutes. ERG b-wave amplitudes were measured from the baseline, or from the a-wave trough when present, to the point of maximum amplitude following this trough. The b-wave implicit time was measured from stimulus onset to the b-wave peak.

Oscillatory potentials (OP) were isolated from the ERG waveforms using digital high-pass filtering with a lower cutoff at 45 to 50 Hz. This cutoff frequency corresponds more closely to the frequency of mouse photopic OP bandwidth^22^ than higher frequencies recommended for humans.^22^,^23^ OP are fast oscillations on the rising phase of the b-wave and originate in inner retinal inhibitory feedback circuits^24^ proximal to the source of the b-wave. They contribute more than 50% of the total photopic ERG energy in humans^25^ and contribute substantially to the ERG changes during light adaptation.^7^ In the mouse photopic ERG, although their contribution to the total power of the dark-adapted ERG is minimal,^22^ they may still influence changes in the smaller photopic response. Therefore, b-waves recorded at 1 minute and 10 minutes of light adaptation were compared before and after subtraction of the isolated OP. The amplitude of the first through the third or fourth OP was measured from its peak to the preceding trough, and the implicit time of the peak was measured from the stimulus onset.

### C-Fos Expression

*C-fos* protein expression in the retinas of light-adapted and dark-adapted WT, *Nrl*^−/−^ and *Rho*^−/−^ mice was used to indicate inner retinal pathway activity. Four-week-old *Rho^−/−^* and 6-month-old *Nrl*^−/−^ mice were compared with age-matched C57BL/6 mice. *Nrl^−/−^* mice were furnished by Anand Swaroop at the National Eye Institute, and all other mice were born and raised in our animal facility. We performed Fos immunolabeling using a rabbit affinity purified polyclonal antibody (sc-52; Santa Cruz Biotechnology, Inc. Dallas, TX, USA). All mice were dark adapted overnight for 16 hours. Light adaptation of unanesthetized WT, *Nrl*^−/−^, and *Rho*^−/−^ mice began between 8 and 10 AM and consisted of a 30-minute exposure to 1.6 log cd·s/m^2^, the same luminance used for recording the light-adapted ERG, followed by 30 minutes of dark. Animals were then euthanized with CO_2_, and eyes were removed and fixed in 4% paraformaldehyde (PFA) in 0.1 M phosphate buffer (pH 7.4) for 30 minutes. Eyes were trimmed to eye-cups and immersed in 4% PFA/PBS for another 1.5 hours. Samples were rinsed and embedded in optimum cutting temperature compound and sectioned at 10-μm thickness. All sections were rinsed in 5% goat serum/PBS, blocked in 20% goat serum/0.5% PBS-T, and then incubated with Fos antibody (1:500) at 4°C overnight. After washing in 5% goat serum/PBS, sections were incubated with Alexa 568 anti-rabbit immunoglobulin (Ig)G/4′,6-diamidino-2-phenylindole (DAPI) (Invitrogen, Carlsbad, CA, USA) for 1 hour, washed in PBS, mounted, and analyzed using a Nikon (Minato, Japan) C2 confocal microscope.

## Results

**Figure 1:**
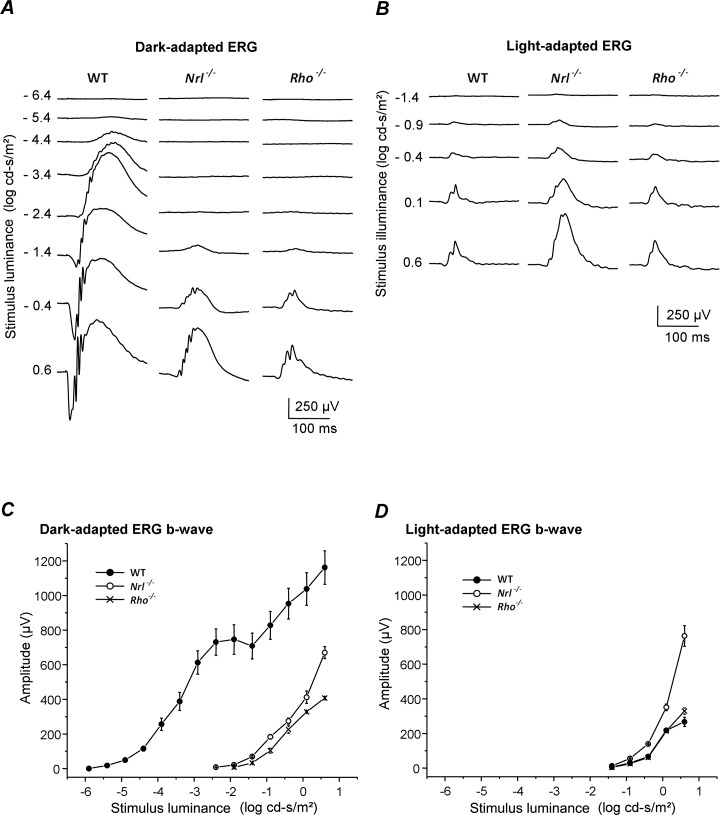
Dark-adapted and light-adapted ERG responses from WT, Nrl^−/−^, and Rho^−/−^ mice across a range of stimulus luminance. (A, C) Fully dark-adapted Nrl^−/−^ and Rho^−/−^ mice lack detectable ERG responses to low-luminance flashes, and the waveforms at higher luminance are much different from WT. (B, D) Waveforms and response-luminance ranges of fully light-adapted Rho^−/−^ and Nrl^−/−^ are similar to their dark-adapted responses and to light-adapted WT. The light-adapted ERG amplitudes from Nrl^−/−^ mice are considerably larger than WT mice, whereas Rho^−/−^ responses are comparable to WT. All values are the mean of three mice ±SE.

Dark-adapted ERG waveforms and luminance-response relationships (Figs. 1A, 1C) were consistent with previous recordings in *Rho*^−/−15,17^ and *Nrl*^−/−16,18^ mice, which demonstrated that these retinas lack functional rods but retain normal cone and cone pathway function. In dark-adapted WT mice, b-waves could be recorded over 6 log units of luminance, whereas the dark-adapted absolute thresholds for both knockout lines were elevated at least 3 log units above WT threshold. Neither line had b-waves in the luminance range for pure rod responses in WT retinas, that is, below the plateau in the WT response representing rod b-wave saturation. Above this luminance, the b-wave in *Rho*^−/−^ and *Nrl*^−/−^ mice increased in parallel with the ERG second rising phase in WT, which is primarily due to cone pathway input.^17^ The dark-adapted b-wave threshold (luminance to give a 50-μV response) of the *Nrl*^−/−^ mice was −1.6 log cd·s/m^2^, only slightly lower than *Rho*^−/−^ mice at −1.3 log cd·s/m^2^, and the amplitude at maximum luminance was almost twice that of *Rho*^−/−^. The dark-adapted waveforms from these two all-cone retinas are easily distinguishable from WT responses by their smaller b-waves and lack of a substantial a-wave (Fig. 1A).

After light adaptation, which suppresses rod responses, the WT and *Rho*^−/−^ ERG luminance-response curves closely resemble each other (Fig. 1B). This is consistent with previous ERG studies in mice at this age and unpublished evidence that *Rho*^−/−^ mice have the same number of cones as WT up to 75 days of age, and the cone ERG amplitude does not begin to decline until 47 days,^17^ when approximately one-half of rod nuclei remain (Bush et al., unpublished observations, 1999). The light-adapted WT and *Rho^−/−^* responses are smaller and faster with larger OPs relative to b-wave size than *Nrl^−/−^* mice, which have supernormal cone b-waves^16^ (Figs. 1B, 1D) due to the presence of many more cones than WT mice.^16^,^18^ The light-adapted cone ERG threshold in WT and *Rho*^−/−^ mice was at a luminance approximately 0.5 log unit higher than for *Nrl*^−/−^mice, and the light-adapted threshold of both the all-cone retinas was approximately 1 log higher, compared with the dark-adapted state, which we previously showed for *Rho*^−/−17^ and *Nrl*^−/−^ mice.^16^

**Figure 2:**
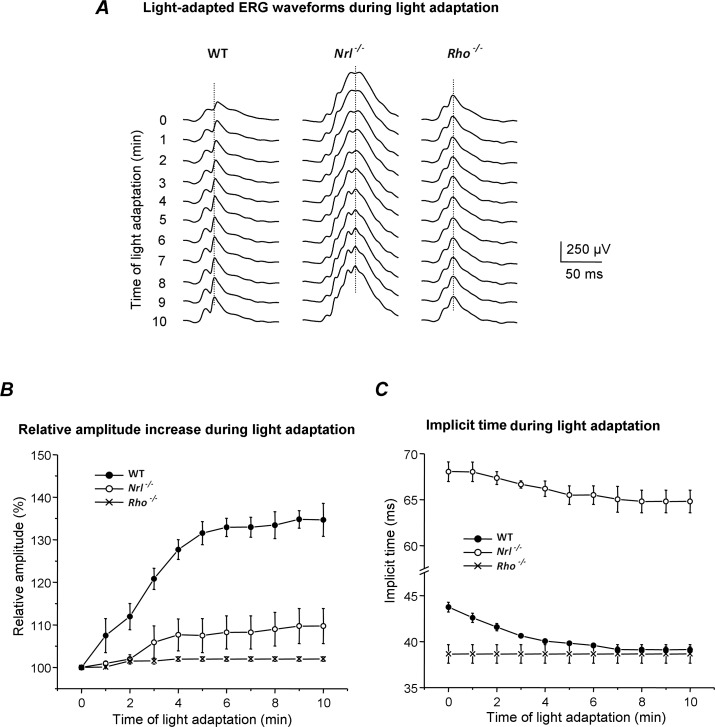
Cone b-wave responses from WT, Nrl^−/−^, and Rho^−/−^ mice to 0.6 cd/m^2^ stimuli during 10 minutes of light adaptation (1.6 log cd/m^2^ steady white background). All mice were dark-adapted overnight. The response at time “zero” was recorded during the first minute of exposure, and subsequent recordings were at 1-minute intervals out to 10 minutes. In WT mice, the amplitudes of the b-wave (including the overlying OPs) increased (B) and the implicit times (C) shortened progressively as the retina adapted to the light. Nrl^−/−^ responses showed a relative amplitude increase and implicit time reduction over the 10 minutes. For Rho^−/−^ mice, neither the amplitude nor implicit time changed across the 10 minutes of light adaptation.

During the first several minutes of light adaptation, the amplitudes of the WT cone b-wave and OP increased, and the implicit times decreased (Fig. 2A). *Nrl*^−/−^ mice showed similar changes, but they were less apparent due to the much larger size of the overall waveform. *Rho^−/−^* waveforms and amplitudes did not change during 10 minutes of light adaptation (Fig. 2A). For WT, measurements on the unfiltered WT b-wave showed a rapid increase in amplitude and decrease in implicit time during the first 5 minutes, followed by a smaller change during the following 5 minutes (Fig. 2B). In WT, the relative increase in amplitude (34.7% ± 3.9%, *n* = 3) over 10 minutes was significantly greater than for *Nrl^−/−^* (9.7% ± 4.1%, *P* = 0.012, *n* = 3) and *Rho^−/−^* mice (2.0% ± 0.5%, *P* = 0.001, *n* = 3). However, the absolute amplitude increase in *Nrl^−/−^* mice (65 ± 27 μV, *n* = 3) was comparable to WT mice (62 ± 8 μV, *n* = 3).

The b-wave implicit time in WT mice (Fig. 2C) was 43.7 ms at the beginning of light adaptation and decreased by 4.6 ± 0.6 ms, *n* = 3 (*P* < 0.05, two-tailed, paired *t*-test) during the light-adaptation period, similar to a previous report.^4^ The b-wave implicit time of *Nrl*^−/−^ mice decreased 3.2 ± 0.6 ms, *n* = 3 (*P* < 0.05) during the 10 minutes of light adaptation. The implicit time in *Rho*^−/−^ mice at 1 minute was identical to that of WT at 10 minutes and did not change significantly during the whole period of light adaptation (0.3 ± 0.3 ms, *n* = 3). In other words, the *Rho*^−/−^ acted as though it were already fully light-adapted as soon as the light was presented at the termination of darkness.

**Figure 3:**
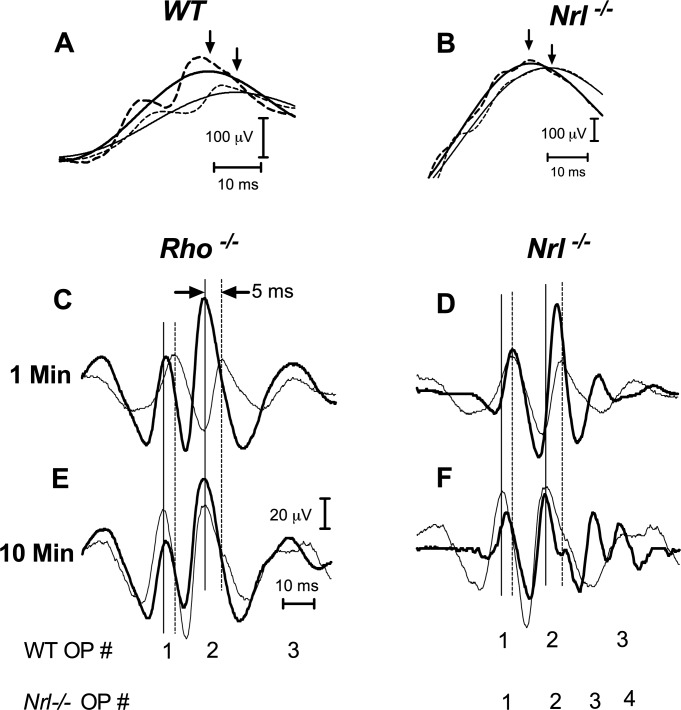
Rho^−/−^, Nrl^−/−^, and WT ERG b-wave and OP changes during light adaptation shown on an expanded time scale. The b-wave leading edge and peak from a representative WT (A) and Nrl^−/−^ mouse (B) before (dashed lines) and after removing the OP (solid lines), recorded during the first minute of light adaptation (thin lines) and after 10 minutes of light adaptation (thick lines). Although the peak times are slightly shorter and amplitudes are larger in WT b-waves with the OP present, the change in amplitude and peak time that occurred during light adaptation remain the same before and after removal of the OP. Note that the amplitude scale bars are different in (A) and (B). (C–F) Isolated OP waveforms from representative Rho^−/−^ (C, E) and Nrl^−/−^ (D, F) mice (thick traces) compared with a WT mouse (thin traces) at 1 and 10 minutes of light adaptation. OP peaks are numbered from the initial trough. The vertical dashed lines mark the time of the first and second OP peaks in the WT mouse during the first minute of light adaptation for comparison to their time at 10 minutes of light adaptation (solid vertical line). The first two OPs in the WT mouse increase in amplitude and decrease in implicit time between 1 and 10 minutes of adaptation. Their timing in the dark-adapted Rho^−/−^ mouse is essentially identical to the light-adapted WT mouse and does not change during light adaptation, whereas the amplitude of the Rho^−/−^ OPs decrease slightly rather than increase. The implicit time of the first two OPs in the Nrl^−/−^ mouse is similar to the WT mouse in both light-adapted and dark-adapted states, whereas the amplitude, which is larger than WT at 1 minute, decreases with further light adaptation. The notch on the trailing side of OP2 and OP3 appear to be additional features of the Nrl^−/−^ light-adapted response. OP4 in Nrl^−/−^ increased in amplitude and decreased in implicit time during light adaptation.

The OP superimposed on the rising phase and peak of the b-wave (Fig. 2A) in WT and *Nrl^−/−^* mice appear to increase in amplitude between 1 and 10 minutes of adaptation. There is an additional late OP in *Nrl^−/−^* that aligns with the b-wave peak, which might contribute to the b-wave timing and amplitude changes. To determine whether this is the case, we isolated the OP by filtering and then subtracting them from the raw waveforms, as described in the methods. Representative b-waves before and after OP removal at 1 and 10 minutes of adaptation in WT and *Nrl^−/−^* are shown on an expanded time scale in Figures 3A, 3B. Removing the OP reduced the WT b-wave amplitude and lengthened the implicit time slightly at both time points (Fig. 3A), but it had no significant effect on the change in amplitude (unfiltered = 51.5 ± 14.2 μV; filtered = 54.6 ± 14.2 μV) or timing (unfiltered = 5.9 ± 1.2 ms; filtered = 5.2 ± 0.4 ms) between 1 and 10 minutes of adaptation. In *Nrl^−/−^* mice, removing the additional OPs that emerged during light adaptation and the OPs on the b-wave crest that increased in prominence had minimal effect on the b-wave implicit time and amplitude (Fig. 3B).

The overall OP waveforms isolated from WT, *Rho^−/−^*, and *Nrl^−/−^* recordings were remarkably similar during the first minute of light adaptation. Two initial larger peaks were followed by a third smaller peak. We used the timing and amplitude of the first two peaks to compare the effects of light adaptation across lines. Figure 3 shows representative examples from each mutant (thick lines) overlaid on a WT example (thin lines) at 1 minute (Figs. 3C, 3D) and 10 minutes (Figs. 3E, 3F) of adaptation. In WT, the OP amplitude was larger and implicit time shorter at 10 minutes than at one minute of adaptation. *Rho^−/−^* OP waveforms are very similar to WT but are larger and faster at the beginning of light adaptation (Fig. 3C). By 10 minutes (Fig. 3E), the implicit times of the first two WT OPs decreased by 4 to 5 ms, and the amplitude approximately doubled. In contrast, both OPs in *Rho^−/−^* mice decreased in amplitude by as much as 25% and showed no timing change at 10 minutes. Thus, the difference between WT and *Rho^−/−^* OP at 1 minute was reduced, and Figure 3E shows they are very similar in amplitude and timing after 10 minutes of adaptation. The third smaller OP behaves similarly: it increases in amplitude and the peak time decreases in WT during light adaptation, whereas only minor change occurred for *Rho^−/−^*. The first two OPs recorded from *Nrl^−/−^* mice have characteristics of both WT and *Rho^−/−^* (Fig 3D): they have the same timing as WT OPs at 1 and 10 minutes, that is, *Nrl^−/−^* OPs decrease in implicit time, as do WT, but, as in *Rho^−/−^*, they are larger than WT at 1 minute and decreased in amplitude during adaptation. There appears to be an additional late OP in *Nrl^−/−^* mice, which peaks at the time of the trough between the second and third WT OP. In Figure 3F, both late OPs increase in amplitude and decrease in implicit time after 10 minutes of adaptation.

**Figure 4:**
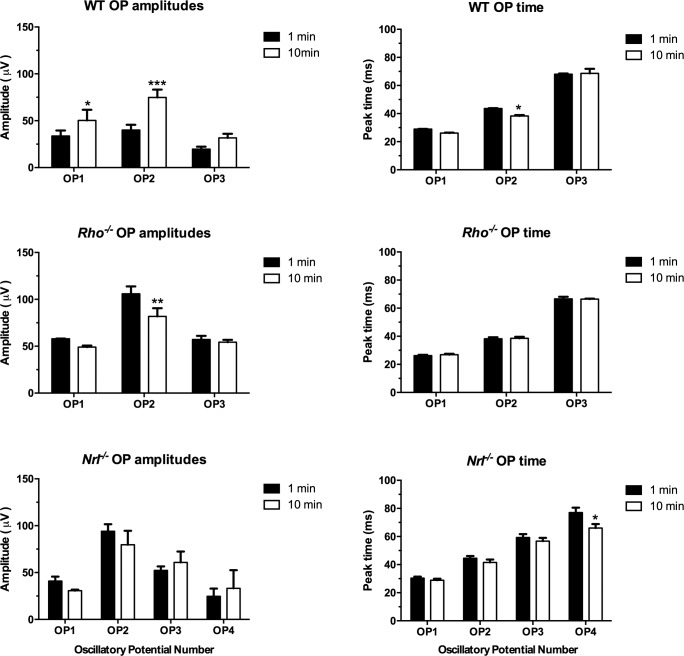
Average amplitude and timing of the oscillatory potentials (OPs 1–3) in WT and Rho^−/−^, and OPs 1 to 4 in Nrl^−/−^ mice. *P < 0.05, **P < 0.01, ***P < 0.005; n = 3 per strain.

The average timing and amplitude changes for OPs 1 to 3 for WT and *Rho^−/−^* mice and OPs 1 to 4 in *Nrl^−/−^* mice are shown in Figure 4 (*n* = 3 for each type). There was a significant increase in the amplitude of OPs 1 and 2 and a decrease in implicit time of OP2 in WT during light adaptation, whereas OP2 amplitude of *Rho^−/−^* decreased to nearly the same level as light-adapted WT with no changes in implicit time. *Nrl^−/−^* mice had no significant changes in the amplitude or timing of OPs 1 to 3, but there was a significant decrease in implicit time of the fourth OP, as shown in the individual example in Figure 3. This example also shows a reduction in the amplitude of OPs 1 and 2 in *Nrl^−/−^*, as also noted for *Rho^−/−^*, and this is reflected in the lower averages in Figure 4. However, the variability was greater in these mice than either WT or *Rho^−/−^*, and the changes were not statistically significant.

**Figure 5:**
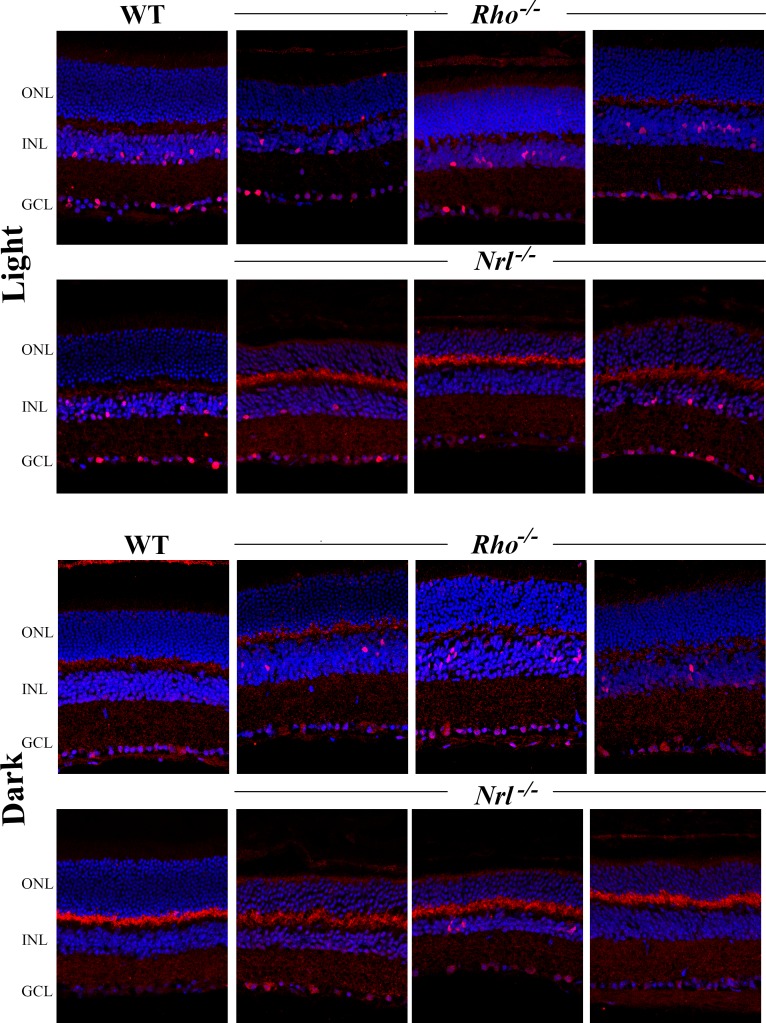
Immunohistochemical staining for c-fos expression in light-adapted and dark-adapted WT, Rho^−/−^, and Nrl^−/−^ retinas. All mice were dark adapted overnight (approximately 16 hours). Light-adapted mice were then exposed to 30 minutes of 1.6 cd·s/m^2^ followed by 30 minutes of darkness. In light-adapted WT retina, many cells in the INL and ganglion cell layer (GCL) stain positive for the Fos protein, but most sections from dark-adapted WT retinas lack positive cells. Light-adapted Rho^−/−^ and Nrl^−/−^ retinas also have Fos-positive cells, although fewer in number than in WT. Staining was more variable across sections from Nrl^−/−^ mice than either WT or Rho^−/−^ and was more consistently found in the periphery than in the central retina. Most sections from dark-adapted WT and Nrl^−/−^ retinas lack Fos-positive cells but the number of stained cells in dark-adapted Rho^−/−^ retinas is similar to light-adapted retinas. Images show representative sagittal sections through the central retinas of two mice for each genotype in each condition. Rho^−/−^ and Nrl^−/−^ were run in separate experiments along with WT mice shown in the same row. Note that there was more diffuse background staining in the outer plexiform layer in the experiment containing WT and Nrl^−/−^ mice.

The *Rho^−/−^* mouse in this study (Fig. 1) and in previous studies^15^,^17^ had normal cone b-wave responses in the fully light-adapted state, indicating normal cone and cone pathway function. However, the data presented here show that these mice, which have a near normal number of rod photoreceptors but lack rod outer segments, do not undergo timing and amplitude changes of the b-wave and OP during the early stages of rod saturation by background light seen in WT mice and even in *Nrl^−/−^* mice in which rods are replaced by cones. This suggests that the *Rho^−/−^* cone ERG amplitude and timing are already in the fully light-adapted physiological state at the beginning of light adaptation. The differences between WT and *Rho^−/−^* in the transition to fully light-adapted state may result from altered rod pathway function proximal to the rods themselves if, for example, the rod pathway in these mice is signaling the equivalent of background light in darkness. To test this hypothesis, we looked at retinal *c-fos* expression in light- and dark-adapted mice (Fig. 5). *C-fos* is an immediate-early gene that undergoes light-induced expression in amacrine and ganglion cells^26^ and is driven by both rod and cone circuits independently.^27^,^28^ After 10 minutes of light adaptation, WT (*n* = 2), *Rho^−/−^* (*n* = 2), and *Nrl^−/−^* mice (*n* = 2) had Fos protein-positive cells in the inner retina, although stained cells were more numerous in WT than either of the mutant strains. Stained cells were more consistently found in the peripheral portions of retinal sections from light-adapted *Nrl^−/−^*, mice but were evenly distributed in WT and *Rho^−/−^* mice. Under dark-adapted conditions, WT (*n* = 2) and *Nrl^−/−^* mice (*n* = 2) had nearly no Fos-positive cells in the inner retina, whereas the number of positive cells in retinas of dark-adapted *Rho^−/−^* mice (*n* = 2) was similar to that see in light-adapted *Rho^−/−^* mice.

## Discussion

This study showed that the cone ERG b-wave of WT mice increases in amplitude and decreases in implicit time during the first 10 minutes of light adaptation, consistent with previous reports in animals^3^,^4^,^29^,^30^ and humans,^5^,^6^,^9^,^31^ as well as the multifocal cone ERG attributed to local responses from the same cell types.^12^ We also show that the OPs, which are thought to originate with inhibitory feedback from the amacrine cells,^24^ change in a similar fashion during light adaptation. To our knowledge, the only published reports on OP changes during light adaptation are for the human ERG.^7^,^32^ As OPs are an integral part of the leading edge and peak of the b-wave, it is possible that much of the b-wave change is actually due to changes in OPs.^7^ Our results show that amplitude changes in the second and largest OP in mice are approximately half that of the b-wave increase during light adaptation, suggesting that OP changes could make a substantial contribution to the b-wave. However, subtracting the OPs from the b-wave by post hoc filtering indicated that both independently show similar timing and amplitude shifts. Thus, it is likely that the changes in both are generated by processes at or distal to the bipolar cell level rather than more proximally in the retina where OPs originate.

Previous studies suggested that the origin of slow changes in the cone ERG during light adaptation, particularly in humans, is the cones themselves.^9^,^30^ However, the idea that rods or rod pathway signaling is the source of these changes was postulated from early studies of the frog retina.^3^ In humans, increased enhancement in the multifocal cone ERG amplitude during light adaptation in regions outside the central retina where the rod density is higher led the authors to propose a role for rods in the enhancement.^12^ In most animal studies, it is difficult to distinguish between a rod and cone pathway source, because adaptation backgrounds that are used to suppress rod responses can also affect cones. Our own previous results in the *Rho^−/−^* mouse showed that backgrounds that completely suppress rods are above the cone dark-adapted threshold and thus affect cone responses.^17^ In addition, a study in ex vivo mouse retina using different levels of background light showed that enhancement of the cone ERG begins in the low to mid mesopic range, at luminances in which both rods and cones are active.^1^ However, the authors were able to show that enhancement of the cone ERG was proportional to the level of rod suppression, and blockade of rod-cone gap junctions mimicked the effect of background light on the cone ERG. Thus, rod-cone gap junctions could provide the mechanism by which rod dark current inhibits cone function, and the enhancement of cone responses during light adaptation represents a release from this inhibition by hyperpolarization of the rods.^1^

In the current study, we used mutant mice that lack functional rods to test in vivo the hypothesis that rods affect the responses of the cone pathway during light adaptation. Previous reports showed that the transducin knockout (*Tr^−/−^*) mouse, which lacks rod phototransduction but has outer segments of near normal length,^33^ has much smaller amplitude and timing changes in the photopic ERG b-wave^14^ and OPs (Schiavone MT, Peachey NS. *IOVS* 2002;43:ARVO E-Abstract 1840) than WT during light adaptation. These results were discussed as in vivo evidence that rods suppress cone function in the dark, and this inhibition is removed when rods are light adapted. However, some enhancement in the cone response was noted between the first and final minute of light adaptation of *Tr^−/−^* mice, suggesting a process intrinsic to the cone pathway also is involved.^14^ This was not the case in *Rho^−/−^* mice in the current study; there was no further decrease in the b-wave or OP timing and no further increase in amplitude during the course of light adaptation. In addition, b-wave and OP timing in *Rho^−/−^* mice during the first minute of adaptation was identical to timing of the b-wave and OP in WT mice reached after only 10 minutes of adaptation. The OP amplitude, which mimics the increase in the b-wave during adaptation, was larger in *Rho^−/−^* at 1 minute than the amplitude of WT OP at 10 minutes, but decreased during adaptation to a level comparable to WT. This is consistent with a previous study that showed that this background luminance level produced an elevation in the cone ERG threshold in *Rho^−/−^* mice.^17^ These results suggest that rods in the 4-week-old *Rho^−/−^* mouse mimic the effect of light adaptation, which is supported by the finding of inner nuclear layer (INL) cells stained with the Fos protein antibody in dark-adapted *Rho^−/−^* retinas (Fig. 5). Neuronal expression of the *c-fos* gene is often used as a marker of inner retinal activation by light.^26^–^28^,^34^ As expected, many cells in the inner retina of light-adapted WT mice were *c-fos* positive, whereas *c-fos* expression was completely absent from dark-adapted WT retina. Taken together, the *c-fos* and ERG findings support two conclusions: (1) rods without outer segments constitutively activate the downstream pathway; (2) cone b-wave changes during light adaptation in WT mice could be attributed solely to activity in the rod pathway.

Unlike *Rho^−/−^* mice, which have rods but no rod outer segments, the retinas of *Nrl^−/−^* mice have no rods, even at a young age. Rods are replaced by normally functioning short-wavelength S-cones,^16^,^18^,^35^ and as a result there are approximately 18 times more cones in *Nrl^−/−^* retina than in WT at 5 weeks of age. In a recent study on older *Nrl^−/−^* mice, cell number and ERG amplitude declined between 1 and 4 months but were relatively stable between 4 and 6 to 8 months at approximately one-half of the 5-week outer nuclear layer (ONL) width and ERG amplitude.^36^ Expression of S- and M-opsin and cone arrestin were maintained at old age, indicating continued presence of cones. Structural evidence in the 18-week-old *Nrl^−/−^* retina shows that these S-cones make synaptic contacts with rod bipolar cells, the second-order neurons in the rod pathway, suggesting that S-cones recruitment of the rod pathway for signaling is maintained.^20^ The large number of S-cones using the rod pathway results in the dark-adapted b-wave being larger than that of the *Rho^−/−^* mouse (Fig. 1C), which has a normal complement of rod (97%) and cone (3.0%) nuclei at 4 weeks (Bush et al., unpublished observations, 1999). During light adaptation, the *Nrl^−/−^* b-wave implicit time decreases and the amplitude increases, comparable to WT mice, although the changes were much smaller relative to the large amplitude and long implicit time of *Nrl^−/−^* mice (Fig. 2). Thus, they could reflect changes in the same (normal) cone pathway response as in WT. Along with the lack of *c-fos* expression in dark-adapted *Nrl^−/−^* retina and a WT-like pattern in light, these results support the idea that the extra cones connected to the rod pathway fulfill the role of rods in modulating cone pathway function during light adaptation. Based on the strong evidence for a role for rod-cone gap junctions in this process,^1^ this implies that the S-cones in the *Nrl^−/−^* retina maintain these gap junctions. To our knowledge, there has been no published work on S-cone gap junction contacts with other cones in *Nrl^−/−^* mice. The preponderance of *c-fos*–stained cells in the peripheral retina we observed in light-adapted *Nrl^−/−^* mice may reflect a gradient of degeneration or perhaps of post synaptic connectivity, because much of the degeneration in old age *Nrl^−/−^* mice was in the INL.^36^

The *Nrl^−/−^* OP response to light adaptation showed more complexity than either WT or *Rho^−/−^* mice with characteristics of both (Figs. 3D, 3F), suggesting they reflect cone-driven contributions from both the rod and cone pathways as indicated by the supernormal b-wave. The timing of the first two OPs was remarkably similar to WT at 1 and 10 minutes of adaptation, but like *Rho^−/−^*, the amplitude of OP2 was higher than WT at 1 minute and decreased at 10 minutes (Fig. 3). These changes were not statistically significant across three mice, however. Thus, changes in the cone pathway OP response in *Nrl^−/−^* mice may be partially masked by rod pathway responses mediated by S-cones. For example, the increased amplitude of OP2 at 1 minute of adaptation may be from S-cones feeding the rod pathway. The reduced amplitude and implicit time after 10 minutes may result from suppressing rod pathway S-cone response with background light and unmasking responses from the cone pathway. A suggestion of this suppressed input may be indicated by the appearance of the small wave on the trailing edge of OP2 at 10 minutes of light adaptation (Fig. 3F). In WT mice, OPs originating in the dark-adapted rod pathway are faster than cone-driven OPs by approximately 13 ms and have five times the total power.^22^ It is remarkable, then, that the initial two OP waveforms in the present study are so similar to WT in timing if they are the combined response from the two pathways. As OP3 in *Nrl^−/−^* in Figures 3D, 3F is not seen in WT, and it did not change significantly during adaptation, it may be a contribution from the additional cones feeding the rod pathway of *Nrl^−/−^* mice. The fourth OP seems to correspond to the third OP in WT. It underwent a statistically significant decrease in implicit time. Perhaps this OP represents the cone pathway response undiluted by contribution from the rod pathway. As such, it is consistent with b-wave changes that suggest WT-like changes in the cone pathway response during adaptation in the *Nrl^−/−^* mouse.

These ERG findings have intriguing implications for physiologic changes to signaling pathways in the mouse retina that result from mutations that alter the fate of developing rod photoreceptors. We hope this will provide an impetus for close anatomic examination of possible structural and synaptic changes in these retinas.
